# The First Telephone

**Published:** 1883-02

**Authors:** 


The First Telephone.—At a recent meeting of the London Physical Society, Prof. Thomson exhibited an early Reis telephone, made by Phillip Reis in 1861, at Frankfort, and designed to transmit speech. It was modeled on the human ear, one form of transmitter being a rudely carved wooden ear with a tympan, having a platinum wire behind hard pressed against a platinum-tipped adjustible spring. Prof. Thomson showed by various proofs that words were actually sent by that and similar apparatus.—*Scientific American.*

